# Multi-Scale Clustering by Building a Robust and Self Correcting Ultrametric Topology on Data Points

**DOI:** 10.1371/journal.pone.0056259

**Published:** 2013-02-12

**Authors:** Hsieh Fushing, Hui Wang, Kimberly VanderWaal, Brenda McCowan, Patrice Koehl

**Affiliations:** 1 Department of Statistics, University of California Davis, Davis, California, United States of America; 2 Animal Behavior Graduate Group, University of California Davis, Davis, California, United States of America; 3 Department of Population Health and Reproduction and California National Primate Research Center, University of California Davis, Davis, California, United States of America; 4 Department of Computer Science and Genome Center, University of California Davis, Davis, California, United States of America; University of Nottingham, United Kingdom

## Abstract

The advent of high-throughput technologies and the concurrent advances in information sciences have led to an explosion in size and complexity of the data sets collected in biological sciences. The biggest challenge today is to assimilate this wealth of information into a conceptual framework that will help us decipher biological functions. A large and complex collection of data, usually called a data cloud, naturally embeds multi-scale characteristics and features, generically termed geometry. Understanding this geometry is the foundation for extracting knowledge from data. We have developed a new methodology, called data cloud geometry-tree (DCG-tree), to resolve this challenge. This new procedure has two main features that are keys to its success. Firstly, it derives from the empirical similarity measurements a hierarchy of clustering configurations that captures the geometric structure of the data. This hierarchy is then transformed into an ultrametric space, which is then represented via an ultrametric tree or a Parisi matrix. Secondly, it has a built-in mechanism for self-correcting clustering membership across different tree levels. We have compared the trees generated with this new algorithm to equivalent trees derived with the standard Hierarchical Clustering method on simulated as well as real data clouds from fMRI brain connectivity studies, cancer genomics, giraffe social networks, and Lewis Carroll's Doublets network. In each of these cases, we have shown that the DCG trees are more robust and less sensitive to measurement errors, and that they provide a better quantification of the multi-scale geometric structures of the data. As such, DCG-tree is an effective tool for analyzing complex biological data sets.

## Introduction

Advances in Information Technology have led to an exponential increase in the amount of data that scientists collect, to the extent that they are now in dire need of new methodologies to summarize and visualize the corresponding large datasets efficiently and rapidly. This is partly the reason that the studies of complex networks, and in particular the identification of community structures within these networks have become a primary focus of research in many fields [1], [2]. Interestingly, this surge in network research in social, biological, physical and mathematical sciences and numerous other fields has also brought a significant surge in the popularity of the hierarchical clustering (HC) algorithm, which was originally proposed more than half a century ago [3]–[5]. The main reasons for the popularity of HC methods are that they are seemingly easy to set up, their computing requirements are usually small, and they provide visual information on data at low costs. As it has become common practice now, a HC tree is constructed on the basis of a choice of a empirical relational measure, either similarity or distance, among object nodes constituting a data cloud of interest, and an ad hoc choice of module, such as complete, single linkage or many others, for prescribing “distances” among sets of nodes [5]. This tree is then conveniently perceived as being able to reveal multi-scale structural information on the data cloud, such as which nodes and which sets of nodes are close to each other. Such a convenient visual apparatus is seemingly bestowed with a “local-to-global” capability. It is not unusual for some scientists to report achieving the ideal ultimate goal of partitioning object nodes into optimally homogeneous clusters in a multi-scale fashion with the HC technique.

Are all these achievements assigned to the HC algorithm “too good to be true”? After being widely used in many scientific areas, indeed confusing questions and doubts in the validity of HC methods have been raised [6], [7]. Despite many such confusions and doubts so far there has been neither satisfactory justifications nor sustainable repudiations for the HC algorithm reported in literature. Nowadays a practitioner is more likely led to place doubts about an incoherent hierarchical clustering tree on his/her own choice of empirical relational measure for the data than on the HC algorithm itself.

Let us start with a review of Hierarchical clustering as it is the method of choice for partitioning data into subsets that share similarities. Starting with an empirical distance or similarity measure 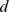, HC proceeds by first merging the two most similar data points. All subsequent steps require a distance between groups of data points. This was solved elegantly by Lance and Williams [8], [9], who proposed a recurrence formula to compute the updated inter cluster distance values that result from the mergers which occur at each level of the procedure. The recurrence formula gives the distance 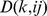 between a data point 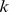 and a cluster 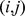 as a function of the empirical distances 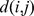, 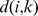 and 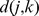:
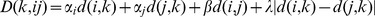
where 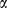, 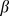, and 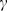 are parameters which define the linkage process.

An interesting property of this recurrence relation is that it usually induces a monotonic hierarchy (i.e. the values in the distance matrix increase monotonically during the agglomerative hierarchical clustering), with the exception of the centroid and median linkage methods [10]. Johnson [5] had shown that an algorithm that produces a monotonic hierarchy also induces a distance metric known as the ultrametric, i.e. that satisfies:
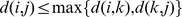
for all triplets 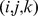, where 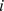, 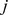, and 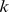 refer to any subsets of the data points. This inequality is clearly stronger that the triangular inequality of a general metric; it has been argued that it should be preserved to capture the true structure of the data set [11].

While most hierarchical clustering algorithms are designed to preserve an ultrametric, they are unfortunately very sensitive to the quality of the empirical distance measure used to compare individual data points. If this empirical distance satisfies the ultrametric inequality, also called strong triangular inequality, HC is expected to perform well. However, it is doubtful that real life data set and distance measure satisfy the ultrametric property exactly. Even if a margin of errors is allowed for each comparison, it was shown that ultrametric hierarchical clustering techniques are not robust with respect to the actual underlying cluster structure in the presence of noise in the empirical distance measure [12].

Noise however is not the only inherent problem of hierarchical clustering. The clustering structure obtained with HC is usually very complex with very many levels. Different choices of the ultrametric, such as complete linkage (i.e. pairwise maximum) or single linkage (i.e. pairwise minimum) often result in different hierarchies. As such, the ultrametric embedded in HC poorly reflects the geometry of the data cloud. Note that this ultrametric is imposed by the method, and not derived from the data. The DCG-tree procedure described in this paper is designed to alleviate this difficulty by letting the empirical distance measure and the data define the ultrametric.

The main argument we make in this paper is that a good partitioning of data into clusters can only be achieved if we have a good understanding of the data geometry and topology [13]. Many clustering techniques have been developed to reach this understanding. Most of those techniques can be formulated as a discrete optimization problem, in which case they involve two distinct steps, namely (i) the definition of some suitable cost function, and (ii), the computation of a partitioning of the data which minimizes this cost function. The number of potentially suitable cost function for clustering is arbitrarily large [14]; in fact, clustering techniques can be classified based on the similarity of their cost functions [15]. Once the cost function is defined, in principle any optimization technique can be used to solve for the optimal partitioning of the data. In practice however, exhaustive approaches are deemed intractable because of the dimensionally of the problems at hand. Many heuristic techniques have therefore been developed (for review, see Puzicha, Hofmann and Buhmann [16]. Among those, it is worth mentioning simulated annealing techniques based on Gibbs sampling [17], deterministic annealing [18], [19], and mean field annealing [20]. These three types of method have in common that they rely on a “temperature” parameter. This parameter can be optimized during the simulation to improve convergence: in the simulated annealing protocol for example, the temperature is gradually lowered, mimicking annealing process in metallurgy. It also provides the algorithm with the possibility to monitor phase transitions (i.e. cluster splits) in order to obtain a meaningful tree topology (see for example Rose [21]).

Transforming the clustering problem into an optimization problem is however not a necessity. We have recently proposed an alternate approach that is inspired from statistical physics, in par with the deterministic annealing and mean field annealing methods mentioned above, that makes use of a temperature parameter to monitor transitions, but that does not explicitly consider a cost function [22]. The main idea of this method is to embed the data geometry into a ferromagnetic potential landscape; its implementation is then based on two key observations. Firstly, it is observed that the empirical distance measure 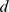 imposes a weighted graph onto the collection of data points (renamed “nodes” in this context). By equating the weight on an edge with a ferromagnetic potential, this weighted graph is seen as equivalent to a potential landscape, typically characterized by many wells with various depths. Secondly, it is possible to explore this landscape and therefore define its geometry by using the popular dynamic Monte Carlo approach. A random walk as a function of “time” will identify the many wells of the potential, as well as the probability of jumping from one well to another. An additional advantage of using dynamic Monte Carlo is that it provides a different dimension to explore the geometry of the landscape, characterized with its temperature parameter 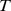. At a high temperature 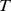, a Markovian walk on the energy landscape will transition from any node to most of the other nodes with more or less equal probabilities. At a low temperature however, the Markov chain tends to get trapped in potential wells for various periods of time depending on the sizes of the well before it can escape. These two observations led to the following two-device algorithm, named Data Cloud Geometry or DCG, for deriving the underlying multi-scale geometry of a data cloud [22]. At a given temperature 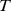, a regulated random walk on the equivalent ferromagnetic landscape as a function of “time” detects information about the number of clusters and the corresponding cluster membership of individual data points. By repeating this procedure at different temperature, the algorithm derives the geometric hierarchy of the data cloud. DCG is similar in spirit to the granular model, which achieve clustering by a sequence of phase transitions on a paramagnetic potential landscape [23], [24]. Its implementation however is simpler and more effective computationally. It has been applied to analyze fMRI data [25], as well as to study binary networks [26].

The DCG procedure originally proposed by Fushing and McAssey [22] is designed to extract unknown geometric information from a data cloud. In this paper we extend this concept and propose to summarize the information collected by DCG in the form of an ultrametric topological space, which is equivalent to a hierarchical tree, the DCG-tree that can also be represented with a Parisi matrix. We validate this approach on simulated and real data for different fields of applications with the corresponding HC-trees. We use these results to illustrate some of the key features of the method, including its robustness with respect to measurement errors, its ability to work on non convex data, and its self-correcting mechanisms. We discuss these results in comparison with similar results obtained with hierarchical clustering. We conclude with a discussion on further developments.

## Methods

### Overview of the DCG-tree procedure

Starting from a set of data points and an empirical measure 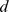 that defines the similarity between these data points, our overall goal is to derive a multi-scale partitioning of these data that illustrates their topology. To address this challenge, we build upon our previous method, Data Cloud Geometry, which gather cluster membership information at different scales, and propose a new algorithmic method that construct an ultrametric topological space from this information, and represent it using either a hierarchical tree or a Parisi matrix. The complete procedure, which we refer to as DCG-tree, includes four main steps, namely:

1. Generate the potential landscape that represents the graph on the data points weighted with the empirical similarity measure,
2. Explore the potential landscape at different temperatures using a Dynamic Monte Carlo procedure to derive its geometry,
3. Build the ultrametric space from the information collected from these multiple Markovian walks,
4. Visualize this ultrametric space using a hierarchical tree or a Parisi matrix.

These five steps are described below. We note that the first two steps have been presented in details in the paper by Hsieh and McAssey [22]; they are outlined here briefly.

### Step 1: Building a potential landscape that mimics the geometry of a data cloud

Consider a 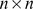 matrix 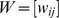, an observed empirical relational matrix of normalized similarity measures on a dataset with 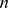 data points, or nodes. 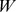 could be a matrix of an absolute value of correlation or simply a transformed distance matrix 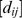 through the transformation 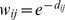 with 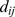 being the corresponding empirical distance between the nodes 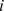 and 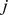. This matrix 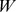 can be represented as a weighted graph 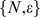 with 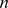 nodes 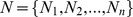 and all possible 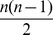 edges 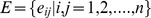 having corresponding weight 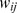.

Given a temperature 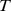, a temperature-regulated potential field 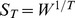 is endorsed on 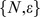. This potential field places potential 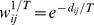 on link 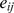, instead of on node 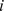 or 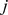. This temperature-regulated potential field can be characterized by the following ratio centered at node 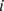: for any 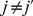,
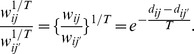
When 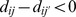, then a very small value of 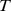 would create a potential well separating links 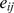 and 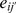. That is, if 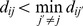, then link 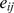 becomes a potential well. This dyad 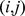 is termed a two-node motif. Similarly motifs of multiple nodes are formed via this idea of potential well.

The definition of the ratio above points to the underlying mechanism that ensures the robustness of DCG-tree. Specifically, when 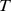 is relatively not too small, the differences 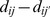
s become less sensitive to 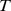, even in the presence of perturbations (or noise). Hence the configuration of the potential wells pertaining to 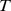 is typically steady. As 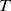 is being raised to a slightly higher value, all potential wells in 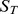 become shallower with a base containing more links, that is, by coupling several motifs into a small cluster. This is the mechanistic dynamics in which a configuration of small clusters is revealed on 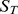.

As 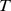 becomes larger, there are fewer potential wells being formed in 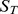 via merging several small clusters. Hence the merging dynamics occurring along the evolution of clustering configurations defines a natural distance among clusters. This indicates that the evolution of potential field 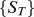 as a function of temperature indeed contains the multi-scale geometric information embedded within 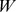.

### Step 2: A re-engineered MCMC method to explore the geometry of the potential landscape

We need to locate on a potential field 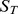 all potential wells and identify their bases' constituents links. This is not an easy task as there hardly exists any visual geometric coordinates for links, and nodes have possibly high dimensional representations. To solve this task, we make use of the characteristics of exceedingly difficult phenomenons when sampling from the Boltzmann distribution via Markov Chain Monte Carlo (MCMC) or dynamic Monte Carlo algorithm at low temperature (see also the Curie-Weiss model [27]). We re-engineer the dynamics of MCMC in order to effectively explore the entire potential field 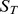.

A Markovian transition probability matrix is calculated as 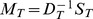 where the degree matrix 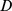 is defined as the diagonal matrix of row-sums 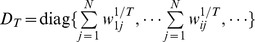. Theoretically an equilibrium trajectory of such a MCMC algorithm based on 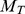 would converge to its stationary probability 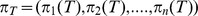 on 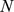 with 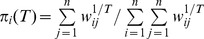 and 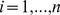. The convergence rate of this MCMC trajectory to 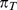 is critically depending on the landscape of the potential field 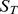. For very large 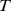, 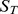 is relatively flat with nearly no or only very shallow potential wells present. In this situation the convergence is very fast and there is only one cluster for all 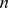 nodes. In contrast, when 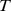 is small, potential wells become deeper and the number of wells becomes large on 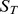. Hence a MCMC trajectory would likely be trapped within a well for a long time before escaping from it. In this case the convergence rate would be very slow and the mixing time could be extremely large for a MCMC trajectory to cover the whole potential field 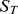.

We note however that we are primarily interested in the composition of potential wells and their base information, and not in 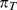. We re-engineer the MCMC algorithm such that it can effectively and exhaustively explore each of every potential well present on 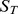 and at the same time extract the base information as motifs or cluster memberships. Here we very briefly review the two key algorithmic devices used in the re-engineered MCMC algorithm, which then called a regulated random walk.

One key algorithmic device is to remove a node after it has been visited for a fixed number of times and modify the transition matrix for the remaining nodes accordingly. Setting the threshold for the number of permitted visits to a given node to be large will result in the Markov chain exploring thoroughly the potential well this node belongs to. But a long visiting time period on every single potential well will add up to a large total computing cost for the whole exploration of the potential landscape. Here it is also understood that one single MCMC exploration does not provide enough creditable geometric information about the landscape at one temperature. Many MCMC explorations on the same landscape at various temperatures have to be performed in order to accumulate and then form reasonably accurate geometric pattern information. Therefore we need to choose the visiting threshold in a way of balancing between a given finite computing budget and a total amount of information content.

The second device is to record the profile of node-removal recurrence time, i.e. the number of successive MCMC steps between two node removals, as the regulated random walk explores 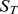. This profile gives rise to a spike of recurrence time whenever a regulated random walk enters a new potential well. Hence nodes removed between two spikes are very likely sharing the same base of a potential well. That is, each regulated random walk trajectory and its profile reveal the membership information for each potential well, either as motifs or clusters. We record this membership information as a 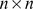 binary matrix with 1 for two nodes sharing a potential well, and 0 otherwise. As we perform an ensemble of such regulated random walks, we generate a collection of 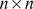 binary potential well sharing matrices, from which we derive a cluster-sharing probability matrix 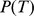.

Such a cluster-sharing probability matrix 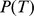 is indeed a summarizing statistic for information on the number of potential wells and their constituting members embedded in the potential field 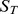. We compute its eigenvalues and set the number of significantly non-zero ones to be the number of potential wells, say 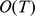. With this information on the number of potential wells, several popular algorithms, such as K-means or spectral clustering, become applicable by using 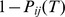 as a distance between the 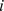
th and 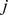
th nodes to extract the constituting base members information. This is the procedure we use for finding the motifs or clusters configuration on a potential field 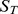 given a temperature 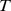.

It is worth mentioning that, to a large extent, the transitivity of cluster membership is built in into this concept of cluster-sharing probability. The cluster-sharing probability matrix becomes a foundation for our DCG algorithm.

### Step 3: Building an ultrametric space from the cluster-sahring probability matrices

We address the issue of finding which, and how many 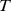
s are needed for computing multi-scale information patterns on the data cloud. In fact we hardly have a priori knowledge on how many focal scales pertain to any given real-world data set or even a simulated one. Hence we apply the algorithmic computations discussed in the previous section on a wide range of 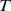 values. The main expectation in our procedure is that at very large 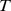 there is only one cluster that includes all nodes. This cluster is very likely a conglomerate. That is, the formation of such a single cluster must come from merging several clusters at a proper temperature according to the potential field perspective. This expectation is carried through as we go further down the merging process.

**Figure 1:**
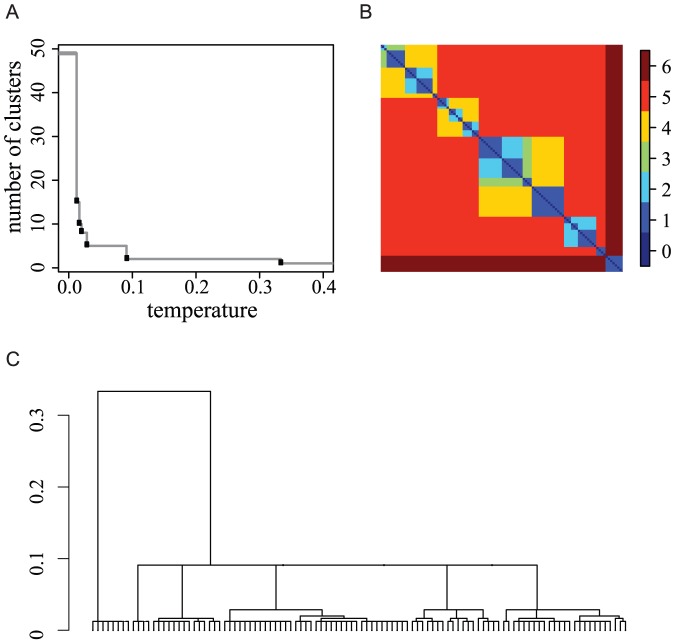
Illustrative DCG-tree based on fMRI data. (A) Plot of the number of clusters vs. temperature, 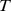; (B) The DCG-Parisi matrix in level numbers of the DCG-tree hierarchy; (C) the DCG-tree.

As 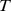 varies from a very small value to very large value, as pointed out in [22], the process of cluster-sharing probability matrix 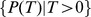 typically evolves through a sequence of phase transitions. We empirically identify such a phase transition sequence by plotting the number of significantly non-zero eigenvalues 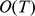 with respect to 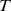. An illustration of such a plot is given in Fig. 1A.

Let us denote the sequence of critical temperatures in increasing order 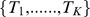 with 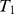 giving rise to a collection of many small motifs and 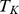 giving rise to one single cluster for all nodes. The data-driven temperatures in the sequence 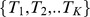 are taken as heights of energy barriers of a ground state to specify an ultrametric upon the data cloud through the following algorithm.

[Ultrametric algorithm on data cloud geometry:] Let 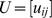 denote the 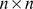 matrix of pairwise ultrametric of the 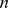 nodes. This matrix is computed as follows:

**A1:** For each pair 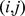 of nodes, we extract its cluster-sharing status sequence as:
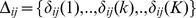
corresponding to the temperature sequence 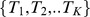, that is, if nodes 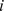 and 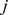 belong to the same motif or cluster of the clustering configuration at temperature 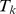, then 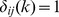, otherwise 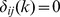, with 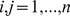 and 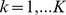;

**A2:** For each 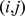 pair, set 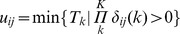.

In [A2], the increasing sequence of temperatures 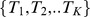 is taken as the free energy barriers separating the potential wells. It is a built-in self-correcting mechanism. We note that in [A1], the cluster-sharing status sequence 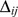 vector may have more than one switch from 0-to-1. When this is the case, the ultrametric between the nodes 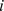 and 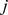 is taken to be the temperature value at which the last 0-to-1 switch occurs, which means that previous identifications are revised for robustness and coherence reasons. This construction can be easily shown to generate an ultrametric topological space.

### Step 4: Representations of the ultrametric topological space

The ultrametric space can easily be represented as a clustering tree with a hierarchy of 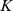 levels. This tree is named the DCG-tree.

This DCG-tree structure has an equivalent matrix representation, which we refer to as the Parisi matrix here. To construct this 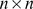 matrix, we arrange its row and column according to the leaves and branches of the DCG-tree. The arrangement is done in such a way that members of each ultrametric ball (i.e. sets of nodes that belong to the same group or cluster) are placed one-by-one on undivided sections along the column and row axes. The ultrametric balls are arranged according to the branching orders, that is, their merging ordering, from the bottom layer toward the top tree layer. Each 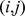 entry of this matrix records the highest energy barrier separating the 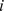 and 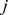 nodes, that is, the ultrametric distance between the two nodes with respect to 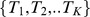. With such an arrangement on the rows and columns, the matrix visually reveals the block-constant structures. We note that the entry recording can take a variety of measures, such as the probability of jumping over an ultrametric distance as used in [28].

## Results

The construction of an ultrametric based DCG-tree as described above differs significantly from the classical construction of a hierarchical clustering (HC) tree. We first illustrate this process on a simple example, as a proof-of-concept. We then analyze the differences between DCG-trees and HC trees on two specifically designed toy problems as well as on three well characterized real data sets. These analyses are designed to provide some answers to the question of why HC trees can be confusing, and how our DCG method can alleviate the corresponding problems.

### An illustrative example

We illustrate the DCG-tree construction based on a real fMRI example. The empirical relational measurement is a wavelet correlation matrix between 106 brain regions of interest (ROIs) from an autistic participant in a neuroscience study [29]. Specifically this correlation matrix contains the pairwise correlation measurements among the 106 dimensional time series derived from the fMRI recording. The DCG-tree is seen as a multiscale summary of extracted functional connectivity patterns among the 106 ROIs. Such brain connectivity patterns can serve as a base for deriving supervised learning tests for diagnosis of autism spectrum disorder [25]. Fig. 1A indicates the existence of 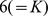 scales, and the 6 clustering configurations are revealed from the Parisi matrix (Fig. 1B) and DCG-tree (Fig. 1C). In the supplemental material, we provide in Figs. S1A and S1B a comparison between this DCG-tree and the HC-trees generated from the same fMRI data.

### Comparing DCG- and HC-tree constructions on simulated data sets

The HC-tree always starts from coupling the dyad with the smallest distance. This starting point is sensitive to any measurement errors, that is, different starting dyad could lead to significantly different tree structures. Two extremes of such structures are related to the choice between two different modules, complete and single linkage, used to conglomerate the clusters as the HC algorithm proceeds.

#### Five Dots Example

**Figure 2:**
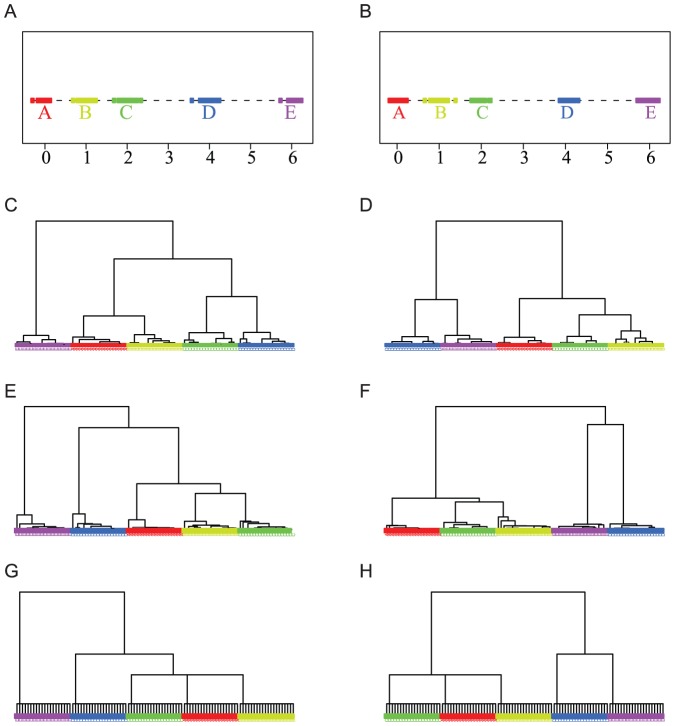
Five Dots Example: HC-tree vs DCG-tree. (A) and (B): Two sets of simulated data under the same setting with five dots as the centers; (C) and (D): HC-trees with complete linkage for data in (A) and (B), respectively; (E) and (F): HC-trees with single linkage for data in (A) and (B), respectively; (G) and (H): DCG-trees for data in (A) and (B), respectively.

Let us consider a simple scenario with five node-centers, A, B, C, D and E, on a straight line with successive distances 1, 1, 1.99 and 2.01. Upon each center on the straight line, 20 independent dots drawn from a normal distribution with standard deviation 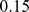 are generated twice (Fig. 2A and 2B). This five-center configuration is specifically designed to represent the “true” data structure, with 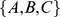 as one single branch (Fig. 2GH).

When the complete module is chosen, there are two equally likely HC-trees that can be generated, depending on the fluctuations in the positions of the five nodes. One tree structure (Fig. 2C) is derived as follows: 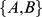 is the starting cluster dyad, then cluster C is pushed to couple with cluster D in the second level. Finally, on the third level, the two cluster dyads 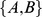 and 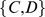 are coupled. The second HC-tree structure (Fig. 2D) is derived as follows: 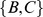 is the starting cluster dyad, cluster A is then coupled with 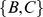 in the second level and finally the cluster dyad 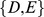 is formed on the third level. The same simulation scenario, but with single linkage, also results into two main tree structures (Fig. 2EF). All four HC-tree structures contains artificial intra- and inter-cluster features compared to the true one. In sharp contrast, the DCG-tree method correctly identifies the true structural triad 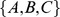 as one single branch. This tree is constructed via the series of critical temperatures 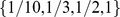 (Fig. 2GH).

#### Two-moon Data Example

Next we turn to a more sophisticated scenario of a data cloud that includes 2000 nodes representing two conformations of the moon, one gibbous and one crescent, with 1000 nodes per conformation.

**Figure 3:**
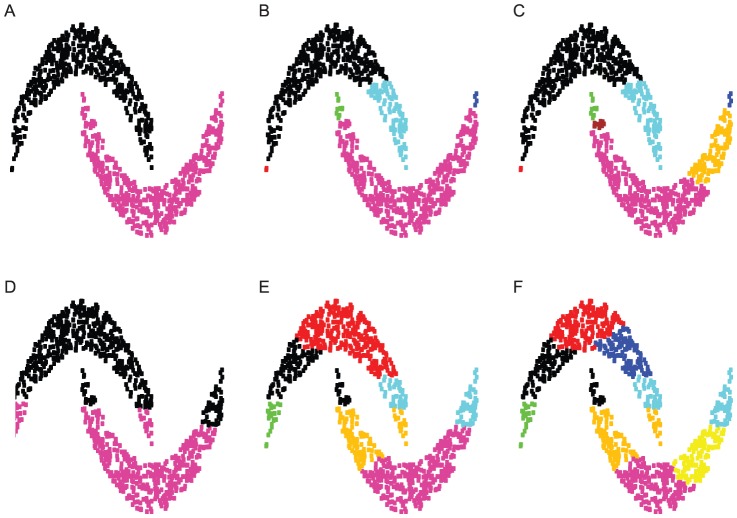
Comparing HC-tree and DCG-tree. (A–C) DCG tree cuts of the two moon data into 2, 6 and 8 clusters, respectively. (D–F) HC tree cuts of the same two moon data into 2, 6 and 8 clusters respectively.

The DCG tree constructed from this data shows three major levels, with 2, 6, and 8 clusters, respectively, and three cluster configurations (Fig. 3ABC, Fig. S2 A). In parallel, we constructed a HC tree from the same data and extracted three different levels from this tree with 2, 6, and 8 clusters (Fig. 3DEF, Fig. S2 B). The three levels of DCG-clustering configurations reveal that each cluster exclusively belongs to one of the two moons; in addition, we clearly observe some self-correction as the algorithm moves from the 8 cluster level to the 6 cluster level. In sharp contrast, many clusters extracted by the HC procedure contain both nodes from the gibbous moon and nodes from the crescent moon. This erroneous behavior of HC is especially evident at the 2-cluster level.

### Comparing DCG- and HC-tree constructions on real data sets

We illustrate several contrasting differences between the DCG and HC trees based on three real data sets. We note that in these cases, the actual geometry of the data is not known; our discussion is therefore more qualitative than quantitative.

#### Functional MRI Data

**Figure 4:**
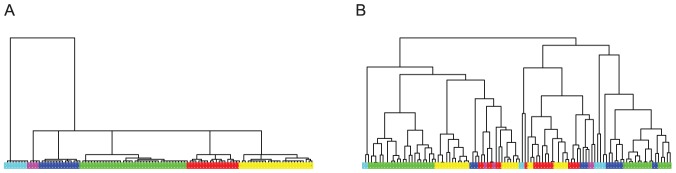
Clustering trees for the 106 ROIs correlation matrix based on fMRI data [**30**]. (A) DCG-tree with coloring based on six-cluster cutoff; (B) HC-tree, colored according to the six clusters of the DCG-tree.

We extend our analysis of the fMRI data example discussed previously. We use nine anatomic brain regions as a reference partitioning on the 106 ROIs [30]. We construct the DCG-tree and the HC-tree (Fig. 4AB). The DCG-tree is color encoded at the level of 6 clusters and the same color coding is mapped onto the HC-tree (Fig. 4B). Clearly, many clusters from the DCG-tree are being scattered in the HC-tree. Assuming that the fMRI data actually capture the characteristics of the anatomic brain regions, we quantified the DCG and HC clusterings against the reference anatomic partitioning using the Rand Index. The DCG-clustering is found to match the anatomic regions well, with a Rand Index of 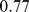, compared to 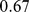 for the HC clustering.

#### Cancer Gene Expression Data

**Figure 5:**
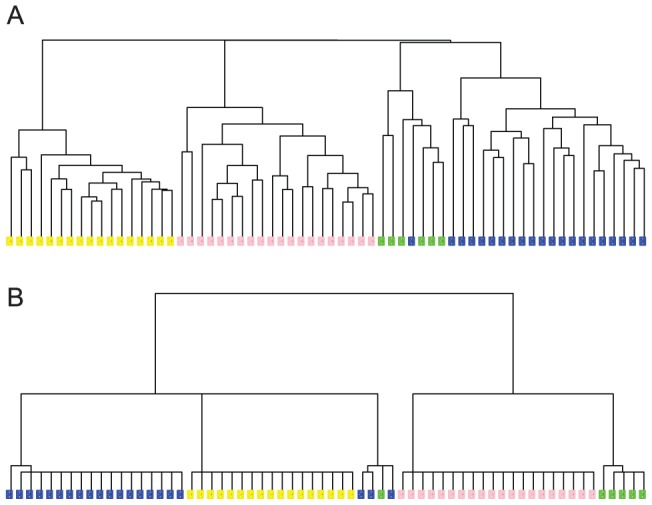
Clustering Trees for the lung cancer data set [**31**] without the AD group. (A) HC-tree; (B) DCG-tree. The color code is: yellow for NL; pink for COID; green for SCLC; blue for SQ (see text for the definitions of the different groups).

**Figure 6:**
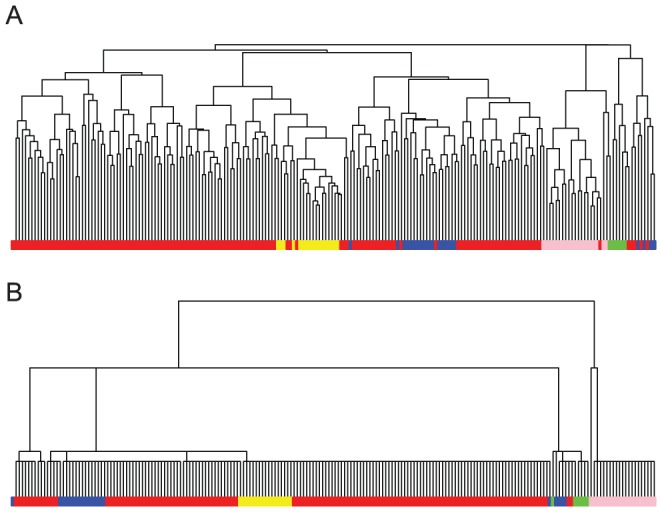
Clustering Trees for the complete lung cancer data set [**31**], including the dominant AD group (in red). (A) HC-tree; (B) DCG-tree. The color code: red for AD; yellow for NL; pink for COID; green for SCLC; blue for SQ (see text for the definitions of the different groups).

Microarray experiments represent a big hope for the diagnosis of cancers as they are expected to enable the measurements of molecular signatures of cancer cells. The main idea is to derive a correspondence between expression patterns of genes and cancer type. To reach this goal, many studies have been published in which gene expression data have been collected from cell lines of patients with known cancer pathologies. Clustering is then performed on these data, with the aim of finding groups of expression patterns that can serve as signatures of the cancer types. Here we re-analyze one such dataset from [31]. This study includes data on 203 patients, out of which 186 were affected by four types of lung cancer, adenocarcinoma (AD, 127 patients), squamous cell lung carcinomas (SQ, 21 patients), pulmonary carcinoids (COID, 20 patients), and small cell lung carcinomas (SCLC, 6 patients), and 17 healthy patients with normal lungs (NL). The original study included expression data for 3,312 genes [31]; out of those 1543 were selected as being the most informative [32]. We note that in this data set, the AD patients represent a very large majority, likely containing many subtypes. This heterogeneity may have adverse effects on the clustering procedure as it could blur the geometric structure of the data. To alleviate this problem, we first removed the AD patients, and constructed DCG- and HC-trees based on the four remaining categories (Fig. 5A and 5B, respectively). These trees then served as seeds to generate the full trees with the AD patients included (Fig. 6A and 6B for the DCG and HC trees, respectively).

Our primary focus is on the three categories NL, COID and SQ, as the smallest category, SCLC contains only 6 patients. We note that the DCG procedure is robust, i.e. the distances between these three categories observed in the small tree and maintained as we move to the larger tree containing all the data points (figures 5A and 6A). On the other hand, the HC procedure does not preserve the geometry of the clusters as more data are included (see figures 5B and 6B). Finally, we note that the DCG-clustering is found to match the known partitions of the full cancer data set well, with a Rand Index of 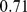, compared to 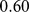 for the HC clustering.

#### Animal behavior: Giraffe social networks

**Figure 7:**
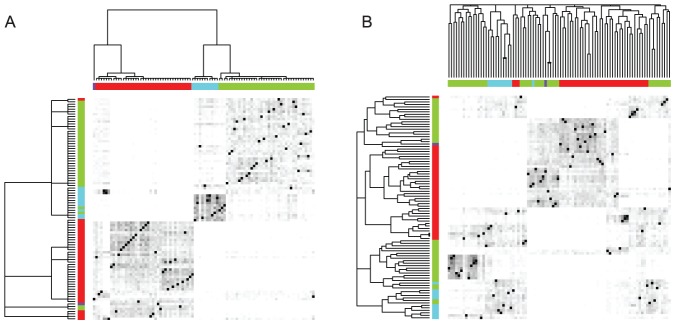
Heatmaps of giraffe social association data. (A) Re-ordered by social data DCG-tree (top axis) and spatial data DCG-tree (left axis); (B) Re-ordered by social data HC-tree (top axis) and spatial data HC-tree (left axis).

Third, we analyze two network datasets showing the spatial patterns and social relationships observed in a population of female giraffe in Ol Pejeta Conservancy, Kenya. The biological question is: to what degree do social and spatial network structures correspond with each other? To address this issue, two DCG-trees are independently constructed for the social and spatial networks. The corresponding heatmaps reveal consistent patterns across social and spatial clustering configurations (Fig. S3 A, C). The spatial and social DCG-trees show not only rather similar hierarchical structures, but also high degrees of correspondence in their clustering configurations, which is visualized via color coding denoting individuals grouped in the same cluster of the social DGC-tree (Fig. 7 A). In contrast, the two HC-trees constructed for the same networks manifest rather different geometries: the spatial one reveals many isolated clustering branches that are inconsistent with the heatmap representation (Fig. S3 D), while the social one shows a structure that is drastically incoherent with the social DCG-tree color coding (Fig. 7 B, Fig. S3 B). See more structural comparisons in the Fig. S4.

The three largest clusters identified in the social DCG-tree correspond to three communities of female giraffes, which occupy somewhat geographically distinct areas of the Conservancy. The eastern red community is spatially and socially separated from the other two by a river. The DCG-tree captures this motif in that the light blue and green communities are closer to each other than to the eastern community, both when the analysis was performed with the social data and the spatial data. However, the HC-tree fails to capture this structural aspect of the data. Further, the social HC-tree groups sub-clusters within the eastern community are as equally distant from each other as from clusters across the river. With both the social and spatial data, the HC-tree also fails to group sub-clusters within the green community as part of the same larger cluster.

#### Linguistics: Lewis Carroll's Doublets network

There is a popular English word game called “Doublets”, which was first introduced by the English author Charles Lutwidge Dodgson (under the pseudonym Lewis Carroll), the author of “Alice's Adventures in Wonderland (1865)”. A network of Doublets can be constructed based on this game. The nodes of this network are set to all English words and a link is created between two nodes if the corresponding words share the same letters, except one (for example, DIVE
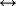
DIRE
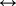
WIRE
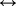
WIPE). Obviously, two words are connected if they have the same length. The whole network is therefore divided into non-connected sub-networks. There are three major connected sub-networks for 7-letter words. Here we consider the smallest one which contains 393 nodes. In order to apply the two clustering algorithms considered here on this sub-network of words, we need a proper measurement distance for all pairs of node. There are many ways to define such a distance measure, as illustrated in one of our previous studies [26]. A very natural measure between two nodes 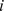 and 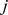 is to consider the sum of edge betweenness along a shortest path linking 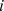 and 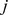, where the “betweenness” of an edge 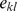 is defined as the number of shortest paths between pairs of nodes that run along 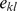. With this definition of a distance, the 7-letter sub-network considered here is transformed into a complete weighted graph.

**Figure 8:**
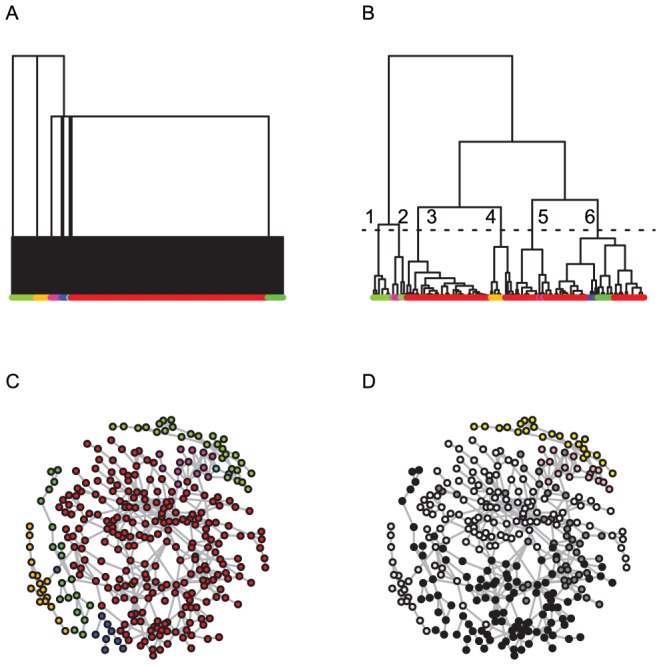
An example from linguistic. Panel (A) shows the DCG-tree of the smallest Doublets sub-network of 7-letter words, that contain 393 nodes (see text for details). Panel (B) shows the corresponding HC-tree, with the leaves colored according to the DCG-tree clustering; six clusters, labeled 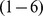, are present when the HC-tree is cut at the level of the dashed line; in panel (C), the network is shown with color markings based on the eight clusters obtained from DCG-tree; finally, in panel (D) the network is shown with colors based on the six clusters labeled in the HC-tree given in panel (D); the color scheme is: 1-yellow, 2-light purple, 3-white, 4-light grey, 5-dark grey, and 6-black.

The DCG-tree of the 7-letter Doublets network, as shown in Fig. 8A, consists of two layers of community structures: one 8-community (with 2 outliers) at a lower temperature and one 3-community at a higher temperature. The composition of these communities usually reveals distinct English word structures with respect to linguistic constraints of phonological rules or even redundancy. It is clear from the computed DCG-tree that its bottom layer contains a dominant community. This community acts like a large magnetic hub that absorbs nearby small communities successively as temperature increases. In contrast, HC clustering does not reveal the presence of this large community, as shown in Fig. 8B.

## Discussion

We have developed a new algorithm that constructs an ultrametric space on a data cloud from the knowledge of an empirical distance measure on the data, and derive an ultrametric tree on this space. This algorithm is based on our previous work on data cloud geometry [22]. Briefly, this algorithm proceeds as follows. The empirical relational measure is transformed into a temperature-regulated potential defined on the links between the nodes. Based on this potential, we extract at very low temperature a collection of motifs, which become building blocks for growing clusters via data-driven merging dynamics as temperature is being raised slowly. A series of phase transitions on this merging dynamics is identified at a series of critical temperatures. These steps are the basis of the DCG procedure described in our previous work [22]. These temperatures are then taken as energy barrier heights to define an ultrametric topology onto the data cloud as it is a system on a ground state. This topology provides measurable and natural distances between clusters. These are the novelties introduced in this paper.

From an information theoretical perspective, the goal of partitioning object nodes into optimally homogeneous clusters is closely related to Kolmogorov's algorithmic sufficiency [33]. On each level of the tree hierarchy, the presence of a cluster indicates that its members uniformly share a typicality. It is known that a perfect partitioning can only be achieved if the properties of the data points are fully captured by a relational measure. It is unfortunately also known that this kind of measure is not likely to be available in real cases. We note that our cluster-sharing probability provides a means for approximating such a typicality, and that the DCG-tree is one step closer to reaching an optimal partitioning of data.

The importance of generating an ultrametric topological structure is related to issues of how to perform randomization or bootstrapping on an observed data cloud. These are pressing issues in biological and many other scientific researches [34], [35]. Ideally any randomization or bootstrapping procedure is meant to generate a surrogate data cloud that is resembling the observed one. An ultrametric tree can serve as the skeleton that has to be maintained in order to sustain the resemblance. That is, the randomization or bootstrapping procedure is applied subject to the constraint of maintaining this skeleton. One effective way of fulfilling this constraint is to work within block-boundaries of Parisi matrix. We are currently working on implementing these ideas.

The two simple toy problems highlight two significant issues with the HC procedure: (i) it is very sensitive to measurement errors and their consequences on distance information and triangular inequalities, and (ii) it is likely to yield artificial intra- and inter-cluster structural information. These two “features” can significantly affect the applicability of the HC method on real world problems. Firstly, it is difficult to be confident in its ability to find motifs that can then be used as building blocks for larger clusters. Secondly, the problems highlighted on this simple test case with a small number of nodes are likely to propagate for much larger data clouds.

The difficulties to extract a robust tree with HC are attached to the concept of distances: the HC procedure relies on an empirical distance measure to detect similarities between nodes in the data; this distance measure is somewhat subjective and very sensitive to measurement errors, as highlighted with the five dot example described above. In addition, the HC procedure needs a distance measure between clusters of nodes. For this, it relies on modules (such as single and complete linkages). These modules are sensitive to measurement errors; in addition, they are also very sensitive to the geometry of the intermediate clusters generated in the merging process. Finally it is important to note that the HC-tree building procedure is deterministic, without any built-in mechanisms for revising previous levels of decision making. A single early mistake can therefore have far reaching effects. Among such effects, we list the creation of many isolated clusters, as observed in Fig. S5A on a real data set. A HC-tree built with the single linkage module is also likely to reveal extreme structural features that grow by including one node at a time, finally resulting in one single branch tree (Fig. S1B and Fig. S5B). This confusing growth pattern seems to be very common, especially when nodes are spread out spatially. This leads to the multi-scale structure information being totally blurred.

We have observed that in comparison, DCG trees are more robust, less sensitive to measurement errors, and provide information on the intrinsic scales embedded within the data cloud under study. We believe that the success of the DCG method is a consequence of two built-in mechanisms. Firstly, the DCG method is designed to replace the empirical distance measure with an effective ultrametric distance that reflects the underlying structure of the data. This is achieved through the characterization of the field potential built on the links in the data (see the description of the DCG method above). This ultrametric is much less sensitive to measurement errors. Secondly, the DCG-tree constructed via procedure [A1] and [A2], has a built-in mechanism to revise previous clustering decisions.

We note that the DCG procedure comes with a high computational cost compared to HC. Let us provide a rough estimate of the computing complexity of DCG. The action of removing nodes one-by-one in the re-engineered MCMC procedure makes the computing cost grow quadratically with respect to the number of nodes 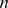 for one single exploration. That is, a single exploration with 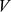 denoting the threshold on the number of permitted visits incurs a computing cost of order 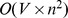. Suppose that we want to build an ensemble of 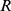 exploration runs at each temperature; the computing complexity for these 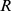 runs is then of order of 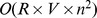. If we decide to make a sequence of 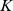 temperatures for the whole geometric information, then the total computing cost for the entire MCMC explorations on 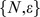 is of order 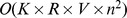. 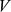, 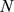 and 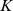 are not independent of 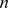: they have to be adjusted to slowly grow as 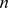 increases. Assuming that at the minimum, this growth is logarithmic, a rough estimate of the computational complexity of our algorithm is therefore of order of 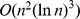. This needs the compared to the complexity of the HC procedure, which is 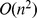). We are currently working on faster implementations of DCG to alleviate this problem.

## Supporting Information

Figure S1 **HC trees of fMRI data.** (A) HC tree with complete linkage; (B) HC tree with single linkage.
(EPS)

Figure S2 **Clustering Trees for Two-moon Data.** (A) DCG Tree; (B) HC Tree with complete linkage.
(EPS)

Figure S3
**Heatmaps of social association and spatial 75** 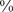 **association female adult giraffe data.** (A) Heatmap of social data based on social DCG tree; (B) Heatmap of social data based on social HC tree; (C) Heatmap of spatial 75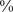 data based on spatial DCG tree; (D) Heatmap of spatial 75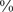 data based on spatial HC tree.(EPS)

Figure S4 **Heatmaps of social association female adult giraffe data.** (A) Heatmap of social data based on social DCG tree (top) and spatial DCG tree (left), colored by spatial DCG tree cut; (B) Heatmap of social data based on social HC tree (top) and spatial HC tree (left), colored by spatial DCG tree cut; (C) Same as (A), displayed as in contrast to (D); (D)Heatmap of social data based on social HC tree (top) and spatial HC tree (left), colored by spatial HC tree cut.
(EPS)

Figure S5 **HC tree of giraffe social association data.** (A) Complete linkage; (B) Single linkage.
(EPS)
